# Annotated Checklist of the Terrestrial Gastropods of Nepal

**DOI:** 10.3897/zookeys.492.9175

**Published:** 2015-03-30

**Authors:** Prem B. Budha, Fred Naggs, Thierry Backeljau

**Affiliations:** 1 Tribhuvan University, Kirtipur, Kathmandu, Nepal; 2 Natural History Museum, Cromwell Road, London, SW7 5BD, UK; 3 Royal Belgian Institute of Natural Sciences, Vautierstraat 29, B-1000, Brussels, Belgium; 4 University of Antwerp, Evolutionary Ecology Group, Groenenborgerlaan 171, B-2020, Antwerp, Belgium

**Keywords:** Gastropoda, checklist, endemic, Nepal

## Abstract

This is the very first checklist of the terrestrial gastropods of Nepal. It includes 138 species and six subspecies, of which 22 species are endemic and four are introduced. It highlights 34 species recorded for the first time in Nepal and provides new distribution records for another 30 species.

## Introduction

The rich diversity of non-marine molluscs of the Indian subcontinent was explored by pioneering 19^th^ century British malacologists. However, as foreigners were restricted from entering Nepal until 1951, the Nepalese malacofauna remained poorly known. *Pupa eurina* Benson, 1864 (now *Pupilla eurina*) may have been the first land snail recorded from Nepal (Budha 2005), but its type locality of ‘Tribeni Ghat’ has not been identified. The earliest confirmed records of terrestrial gastropods from Nepal were an unidentified *Nanina* species and *Anadenus* sp. [?= *Anadenus giganteus* Heynemann] from Kathmandu Valley (Nevill 1878: p. 27 and 65 respectively). No publications on Nepalese snails appeared for the following 95 years until the description of two new species and two new subspecies of *Hemiphaedusa* by Nordsieck (1973) and a chromosomal study on some ariophantids (Kiauta and Butot 1973). Subsequently, Schileyko and Frank (1994) described a new species (*Laevozebrinus nepalensis*), a new genus (*Nepaliena*) and a new enid subfamily (Pseudonapaeinae) from hills surrounding Kathmandu Valley. In addition, they described the reproductive anatomy of *Oxytesta orobia* (Benson, 1848). Since then, several occasional papers on the Nepalese terrestrial gastropods have been published (Kuznetsov 1996, Kuznetsov and Schileyko 1997, 1999, Schileyko and Kuznetsov 1998a, 1998b, Raut 1999, Schileyko 1999, Wiktor 2001a, Subba and Ghosh 2001, 2008, Wiktor and Bössneck 2004, Budha 2005, Kuzminykh and Schileyko 2005, Bössneck 2006, Budha and Naggs 2008, Gerber and Bössneck 2009, Budha et al. 2012, Schileyko and Balashov 2012, Khanal and Budha 2013). Despite the fact that the study of Nepalese terrestrial gastropods is still in its infancy, there is a need for at least a provisional checklist as a starting point for further study. The present paper aims at providing such a list.

The data included here are based on published records and field investigations from 2006–2010 by Prem Budha. Collected material has been deposited in the Central Department Zoology Museum of Tribhuvan University (CDZMTU), Kirtipur, Kathmandu, Nepal. The list provides taxonomic notes where needed, as well as distribution ranges of genera and species. The original names of the type species of genera and subgenera are provided. An attempt was made to standardize the use of geographical place names and local features but, owing to the nature of this data, it was not always possible to do so. The district name is mentioned for all species from Nepal with particular locations such as hill, forest, and village names wherever data are available. National park or conservation areas are given without district names because most national parks extend across more than one district. Particular locations within national parks are given where known. Indian states are given with particular location(s) wherever data are available. The systematic arrangement at family and more inclusive levels is based on Bouchet and Rocroi (2005). Family names are arranged according to Bouchet and Rocroi (2005), while genus and species names are arranged alphabetically. The list includes 138 species and six subspecies, including 22 endemic species, four introduced species, 34 new species for Nepal, and new distribution records for 30 species.

## Systematics

Class: Gastropoda Cuvier, 1795

Clade: Caenogastropoda Cox, 1960

Superfamily: Cyclophoroidea J.E. Gray, 1847

Family: Cyclophoridae J.E. Gray, 1847

Subfamily: Cyclophorinae J.E. Gray, 1847

Genus: *Cyclophorus* Montfort, 1810^1^

Distribution: Subtropical and tropical Asia (Gude 1921, Benthem Jutting 1948, Zilch 1956, Kongim et al. 2006).

Type species: *Helix volvulus* O.F. Müller, 1774

Subgenus: *Glossostylus* Kobelt & Möllendorff, 1897

Distribution: India; Sri Lanka; Myanmar; Thailand; Vietnam; Taiwan; Malaysia; Philippines (Gude 1921).

Type species: *Cyclostoma validum* Sowerby, 1842

Cyclophorus (Glossostylus) fulguratus (L. Pfeiffer, 1852)^2^

Distribution: Myanmar; Thailand; Vietnam (Gude 1921).

Nepal: Ilam, Jhapa, Morang, Sunsari, Dharan, Udayapur and Gulmi Districts (Subba and Ghosh 2001).

Subgenus: *Kobeltostylus* Egorov, 2006^3^

Distribution: Bangladesh; India; Sri Lanka; Myanmar; Philippines (Gude 1921).

Type species: *Helix involvulus* O.F. Müller, 1774

Cyclophorus (Kobeltostylus) pyrotrema Benson, 1854

Distribution: Bangladesh; India; Myanmar (Gude 1921).

Nepal: Lalitpur District-Phulchowki Hill (Kuznetsov and Schileyko 1997).

Subgenus: *Annularia* Schumacher, 1817^4^

Distribution: India; Sri Lanka; Myanmar; Philippines (Gude 1921).

Type species: *Annularia aurantiaca* Schumacher, 1817

Cyclophorus (Annularia) aurantiacus (Schumacher, 1817)^5^

Distribution: Thailand; Myanmar; W Malaysia (Nevill 1878, Gude 1921).

Nepal: Ilam, Morang, Sunsari, Dharan and Udayapur Districts (Subba and Ghosh 2001).

Genus: *Theobaldius* Nevill, 1878

Distribution: Sri Lanka; S and NE India; Myanmar (Gude 1921).

Type species: *Cyclophorus annulatus* L. Pfeiffer, 1847

*Theobaldius* sp.

New species record for Nepal: Shivapuri-Nagarjun and Langtang National Parks.

Genus: *Scabrina* W.T. Blanford, 1863^6^

Distribution: S and SE Asia (Gude 1921, Maassen 2006).

Type species: *Cyclophorus calyx* Benson, 1847.

*Scabrina phaenotopicus* (Benson, 1851)

Distribution: India: W Bengal-Darjeeling, Sikkim (Gude 1921).

Nepal: Raheem et al. (2010).

New distribution records from Nepal: Chitwan National Park, Tanahu District-Shiddha Cave area and Lalitpur District-Phulchowki Hill.

Genus: *Pterocyclos* Benson, 1832

Distribution: India; Sri Lanka; SE Asia (Raheem and Naggs 2006, Ramakrishna et al. 2010, Kongim et al. 2013).

Type species: *Pterocyclos rupestris* Benson, 1832

Pterocyclos cf. brahmakundensis Godwin-Austen, 1915

Distribution: India: Assam-Brahmakund (Gude 1921).

New species record for Nepal: Langtang National Park.

Subfamily: Alycaeinae W.T. Blanford, 1864^7^

Genus: *Alycaeus* J.E. Gray, 1850

Distribution: India; Nepal; Myanmar; China; Japan; Taiwan; Korea; Thailand; Vietnam; Laos; Philippines; Indonesia; Malaysia; Australia (Gude 1921,Tarruella and Domènech 2011).

Type species: *Alycaeus eydouxi* Venmans, 1956^8^

Subgenus: *Alycaeus* J.E. Gray, 1850

Distribution: India; Myanmar; China; Malaysia; Japan (Gude 1921).

Type species: *Alycaeus eydouxi* Venmans, 1956

Alycaeus (Alycaeus) burti Godwin-Austen, 1874

Distribution: India: Arunachal Pradesh, Assam, Mizoram-Akha Hills, Dihiri Parbat; Bhutan (Gude 1921, Ramakrishna et al. 2010).

Nepal: Solukhumbu District (Kuznetsov and Schileyko 1997).

New distribution records from Nepal: Kathmandu District-Champadevi Hill, Lalitpur District-Phulchowki Hill and Shivapuri-Nagarjun National Park.

Alycaeus (Alycaeus) lohitensis Godwin-Austen, 1914

Distribution: India: Assam, Arunachal Pradesh (Gude 1921, Ramakrishna et al. 2010).

Nepal: Lalitpur District-Phulchowki Hill (Kuznetsov 1996).

Alycaeus (Alycaeus) yamneyensis Godwin-Austen, 1914

Distribution: India: Arunachal Pradesh-Yamne Valley, Abor Hills (Gude 1921).

Nepal: Tarruella and Domènech (2011).

Genus: *Chamalycaeus* Kobelt & Möllendorff, 1897

Distribution: India; Nepal; Myanmar; China; Taiwan; Korea; Thailand; Vietnam; Laos; Philippines; Indonesia; Malaysia; Australia (Gude 1921, Tarruella and Domènech 2011).

Type species: *Alycaeus andamaniae* Benson, 1861

Subgenus: *Dicharax* Kobelt & Möllendorff, 1900

Distribution: NE India; Myanmar; China; Malaysia (Gude 1921).

Type species: *Alycaeus hebes* Benson, 1857

Chamalycaeus (Dicharax) bicrenatus (Godwin-Austen, 1874)

Distribution: NE India: Assam, Nagaland-Naga Hill (Ramakrishna et al. 2010).

Nepal: Lalitpur District-Phulchowki Hill (Kuznetsov 1996).

Chamalycaeus (Dicharax) digitatus (H.F. Blanford, 1871)

Distribution: NE India: W Bengal-Darjeeling, Sikkim-Richila Peak; W Bhutan (Gude 1921).

New species record for Nepal: Kathmandu District-Champadevi Hill, Lalitpur District-Phulchowki Hill, Shivapuri-Nagarjun National Park.

Chamalycaeus (Dicharax) inflatus (Godwin-Austen, 1874)

Distribution: NE India: Nagaland-Naga Hills (Ramakrishna et al. 2010).

Nepal: Shivapuri-Nagarjun National Park-Nagarjun Forest (Khanal and Budha 2013).

Chamalycaeus (Dicharax) notatus (Godwin-Austen, 1876)

Distribution: NE India: Nagaland-Naga Hills, Arunachal Pradesh-Dafla Hills (Gude 1921).

Nepal: Solukhumbu District (Kuznetsov and Schileyko 1997).

Chamalycaeus (Dicharax) plectochilus (Benson, 1859)

Distribution: NE India: W Bengal-Darjeeling, Sikkim-Damsang Peak; W Bhutan (Gude, 1921).

New species records for Nepal: Kathmandu District-Champadevi Hill, Shivapuri-Nagarjun and Langtang National Parks.

Chamalycaeus (Dicharax) strangulatus (L. Pfeiffer, 1846)^9^

Distribution: This is the only species of Chamalycaeus (Dicharax) recorded from the W Himalaya, NW India: Himachal Pradesh-Simla, Uttarakhand-Kumaon, Nainital (Ramakrishna et al. 2010).

New species record for Nepal: Shivapuri-Nagarjun National Park.

Chamalycaeus (Dicharax) stylifer (Benson, 1857)

Distribution: NE India: W Bengal-Darjeeling and Sikkim; Bhutan (Gude 1921).

New species records for Nepal: Lalitpur District-Phulchowki Hill, Shivapuri-Nagarjun and Langtang National Parks.

Subgenus: *Cycloryx* Godwin-Austen, 1914

Distribution: NE India to Myanmar (Gude 1921).

Type species: *Alycaeus constrictus* Benson, 1851

Chamalycaeus (Cycloryx) otiphorus (Benson, 1858)

Distribution: NE India: W Bengal-Darjeeling, Sikkim-Pankhabari, Meghalaya, Nagaland (Gude 1921, Ramakrishna et al. 2010).

Nepal: Lalitpur District-Phulchowki Hill (Kuznetsov 1996).

New distribution record from Nepal: Shivapuri-Nagarjun National Park.

Chamalycaeus (Cycloryx) summus (Godwin-Austen, 1914)

Distribution: NE India: Sikkim-Richila Peak; W Bhutan (Gude 1921).

Nepal: Solukhumbu District (Kuznetsov and Schileyko 1997).

Family: Diplommatinidae L. Pfeiffer, 1856

Subfamily: Diplommatininae L. Pfeiffer, 1856

Genus: *Diplommatina* Benson, 1849^10^

Distribution: India; Nepal; China; Indonesia; Vietnam; Singapore; Malaysia; Japan; Philippines; Taiwan; Papua New Guinea; Fiji (Gude 1921, Zilch 1953, Schileyko and Kuznetsov 1997, Maassen 2002, Panha and Burch 2005, Webster et al. 2012).

Type species: *Bulimus folliculus* L. Pfeiffer, 1846

Subgenus: *Diplommatina* Benson, 1849

Distribution: N India; Nepal; China; Malaysia; Philippines; Japan; Taiwan; Papua New Guinea; Fiji (Gude 1921, Zilch 1953, Schileyko and Kuznetsov 1997, Panha and Burch 2005, Webster et al. 2012).

Type species: *Bulimus folliculus* L. Pfeiffer, 1846

Diplommatina (Diplommatina) exserta Godwin-Austen, 1886

Distribution: Myanmar: Damotha Cave, etc., Moulmein, now Mawlamyine (Gude 1921).

New species record for Nepal: Tanahu District-Siddha Cave area.

Diplommatina (Diplommatina) folliculus (L. Pfeiffer, 1846)

Distribution: NW India: Himachal Pradesh-Landour, Simla, Uttarakhand-Nainital (Naggs 1997, Ramakrishna et al. 2010).

New species records for Nepal: Lalitpur District-Phulchowki Hill, Shivapuri-Nagarjun and Langtang National Parks.

Diplommatina (Diplommatina) munipurensis Godwin-Austen, 1892

Distribution: NE India: Manipur; Myanmar (Gude 1921).

New species records for Nepal: Lalitpur District-Phulchowki Hill, Langtang National Park.

Diplommatina (Diplommatina) oviformis Fulton, 1901

Distribution: India: W Bengal-Darjeeling (Gude 1921).

Nepal: Solukhumbu District (Kuznetsov and Schileyko 1997).

New distribution records from Nepal: Hills surrounding Lalitpur and Kathmandu Districts, Shivapuri-Nagarjun and Langtang National Parks.

Diplommatina (Diplommatina) pachychilus Benson, 1857

Distribution: NE India: W Bengal-Darjeeling (Gude 1921).

Nepal: Solukhumbu District (Kuznetsov and Schileyko 1997).

New distribution records from Nepal: Shivapuri-Nagarjun and Langtang National Parks.

Diplommatina (Diplommatina) regularis Fulton, 1901.

Distribution: NE India: W Bengal-Darjeeling (Ramakrishna et al. 2010).

New species record for Nepal: Shivapuri-Nagarjun National Park-Baghdwar.

Diplommatina (Diplommatina) silvicola Godwin-Austen, 1886

Distribution: NE India: Assam-North Cachar, Jenta Hajuma Peak (Gude 1921, Ramakrishna et al. 2010).

New species record for Nepal: Shivapuri-Nagarjun National Park-Balaju, Pani Tanki.

Diplommatina (Diplommatina) sperata W.T. Blanford, 1862

Distribution: Myanmar (Gude 1921).

Nepal: Solukhumbu District (Kuznetsov and Schileyko 1997).

Subgenus: *Metadiancta* Möllendorff, 1898

Distribution: NE India: Assam, Manipur, Nagaland; Myanmar; Vietnam (Gude 1921).

Type species: *Diplommatina dohertyi* Godwin-Austen, 1892

Diplommatina (Metadiancta) miriensis Godwin-Austen, 1917

Distribution: NE India: Arunachal Pradesh-Miri Hills (Gude 1921).

New species records for Nepal: Shivapuri-Nagarjun and Langtang National Parks.

Subgenus: *Sinica* Möllendorff, 1885

Distribution: India; Nepal; Myanmar; China; Japan; Philippines; Indonesia; Malaysia; Papua New Guinea; Taiwan (Gude 1921, Zilch 1953, Kuznetsov and Schileyko 1997).

Type species: *Diplommatina collarifera* Schmacker and Boettger, 1877

Diplommatina (Sinica) canarica Beddome, 1875^11^

Distribution: India: Western Ghats, Karnataka, Maharashtra (Ramakrishna et al. 2010, Raheem et al. 2014).

Nepal: Solukhumbu District (Kuznetsov and Schileyko 1997).

Family: Pupinidae L. Pfeiffer, 1853

Subfamily: Pupininae L. Pfeiffer, 1853

Genus: *Schistoloma* Kobelt, 1902

Distribution: Indian Himalaya; Nepal; China; Thailand; W Malaysia; Sumatra; Borneo; Philippines (Gude 1921, Bartsch 1915, Tumpeesuwan and Panha 2008).

Type species: *Cyclostoma altum* Sowerby, 1842

Schistoloma cf. funiculalum (Benson, 1838)^12^

Distribution: India: W Bengal-Darjeeling (Gude 1921).

New species records for Nepal: Lalitpur District-Phulchowki Hill, Shivapuri-Nagarjun and Langtang National Parks.

Superfamily: Ellobioidea L. Pfeiffer, 1854 (1822)

Family: Ellobiidae L. Pfeiffer, 1854 (1822)

Subfamily: Carychiinae Jeffreys, 1830

Genus: *Carychium* O.F. Müller, 1773

Distribution: Very widely distributed from N and C America, Europe to S and SE Asia (Burch and Panha 2002, Thompson 2011).

Type species: *Carychium minimum* O.F. Müller, 1774

*Carychium minusculum* Gredler, 1888^13^

Distribution: China "aus Hope" (Gredler 1888).

Nepal: Langtang National Park-Syabru (Kuznetsov and Schileyko 1997), Kavre District-Chandeshwari (Nesemann et al. 2007).

*Carychium* sp.^14^

New species records for Nepal: Lalitpur District-Phulchowki Hill, Shivapuri-Nagarjun and Langtang National Parks.

Clade: Systellommatophora Pilsbry, 1948

Superfamily: Veronicelloidea J.E. Gray, 1840

Family: Veronicellidae J.E. Gray, 1840

Genus: *Laevicaulis* Simroth, 1913

Distribution: Pantropical (Stanisic 1998).

Type species: *Vaginulus alte* Férussac, 1822

*Laevicaulis alte* (Férussac, 1822)^15^

Distribution: The geographical origin of *Laevicaulis alte* is uncertain, but it has been widely distributed in tropical and subtropical countries through human agency (Stanisic 1998).

New distribution records from Nepal: Widely distributed throughout most districts of Tarai and inner Tarai.

Clade: Stylommatophora Schmidt, 1855

Superfamily: Succineoidea H. Beck, 1837

Family: Succineidae H. Beck, 1837

Subfamily: Catinellinae Odhner, 1950

Genus: *Quickia* Odhner, 1950

Distribution: W and E Africa; Mascarene Islands; Seychelles; Aldabra; India; Nepal (Patterson 1975, Schileyko 2007).

Type species: *Succinea concisa* Morelet, 1848

*Quickia* sp.

New species record for Nepal: Chitwan District-Sauraha (collected from flower vase at hotel).

Subfamily: Succineinae H. Beck, 1837

Genus: *Succinea* Draparnaud, 1801

Distribution: Nearly circumglobal (Schileyko 2007), the Northern Hemisphere; Australia; some Pacific islands (Thompson 2011).

Type species: *Succinea amphibia* Draparnaud, 1801 (= *Helix putris* Linnaeus, 1758)

*Succinea* sp.

New species records for Nepal: Kathmandu and Lalitpur Districts.

Superfamily: Pupilloidea Turton, 1831

Family: Pupillidae Turton, 1831

Subfamily: Pupillinae Turton, 1831

Genus: *Pupilla* Fleming, 1828

Distribution: Temperate N America; Europe; Africa; Asia; Australia (Gude 1914, Pokryszko et al. 2009).

Type Species: *Pupa marginata* Draparnaud, 1801(= *Turbo muscorum* Linnaeus, 1758).

*Pupilla annandalei* Pilsbry, 1921^16^

Distribution: Pakistan.

Nepal: Pokryszko et al. (2009).

*Pupilla eurina* (Benson, 1864)^17^

Distribution: Endemic to Nepal.

Nepal: Tribeni Ghat (Blanford and Godwin-Austen 1908), Annapurna Conservation Area-Tukuche (Kuznetsov and Schileyko 1997).

New distribution record from Nepal: Langtang National Park-Gosainkund.

*Pupilla triplicata* (Studer, 1820)

Distribution: Europe and C Asia (Sysoev and Schileyko 2009).

Nepal: Annapurna Conservation Area-Tukuche (Kuznetsov and Schileyko 1997).

Family: Pyramidulidae Kennard & Woodward, 1914

Genus: *Pyramidula* Fitzinger, 1833

Distribution: Holarctic and S Asia (Gittenberger and Bank 1996, Schileyko and Balashov 2012).

Type species: *Helix rupestris* Draparnaud, 1801

*Pyramidula humilis* (Hutton, 1838)^18^

Distribution: NW India: Himachal Pradesh, Punjab, Uttarakhand (Ramakrishna et al. 2010)

Nepal: Shivapuri-Nagarjun National Park-Nagarjun Forest (Khanal and Budha 2013).

*Pyramidula kuznetsovi* Schileyko & Balashov, 2012^19^

Distribution: Endemic to Nepal.

Nepal: Mustang District-Muktinath (Schileyko and Balashov 2012).

Family: Valloniidae Morse, 1864

Genus: *Vallonia* Risso, 1826

Distribution: Holarctic (Gerber and Bössneck 2009).

Type species: *Vallonia rosalia* Risso, 1826 (= *Helix pulchella* O.F. Müller, 1774)

*Vallonia costohimala* Gerber & Bössneck, 2009

Distribution: Endemic to Nepal.

Nepal: Northern districts from Darchula to Panchthar (Gerber and Bössneck 2009).

*Vallonia himalaevi* Gerber & Bössneck, 2009

Distribution: Endemic to Nepal.

Nepal: Northern districts from Darchula to Panchthar (Gerber and Bössneck 2009).

*Vallonia kathrinae* Gerber & Bössneck, 2009

Distribution: Endemic to Nepal.

Nepal: Mugu and Mustang Districts (Gerber and Bössneck 2009).

*Vallonia ladacensis* (Nevill, 1878)

Distribution: India: Western Ghats, Jammu and Kashmir; Nepal; Tibet; Tianshan Turkey (Gerber and Bössneck 2009, Raheem et al. 2014).

Nepal: Bajura, Darchula, Humla and Mustang Districts (Gerber and Bössneck 2009).

*Vallonia tenuilabris* (A. Braun, 1843)

Distribution: Kazakhstan; Tajikistan; NW India: Jammu and Kashmir; Tibet; Siberia; N China; Mongolia to Russia (Gerber and Bössneck 2009).

Nepal: Solukhumbu and Taplejung Districts (Gerber and Bössneck 2009).

Family: Vertiginidae Fitzinger, 1833

Subfamily: Vertigininae Fitzinger, 1833

Genus: *Truncatellina* Lowe, 1852

Distribution: Holarctic (Schileyko 1998).

Type species: *Pupa linearis* Lowe, 1852

*Truncatellina* sp.

Nepal: Annapurna Conservation Area-Khobang (Kuznetsov and Schileyko 1997).

Subfamily: Gastrocoptinae Pilsbry, 1918

Genus: *Gastrocopta* Wollaston, 1878

Distribution: Almost cosmopolitan extending to all tropical and warm temperate continents but extinct in Europe (Pilsbry 1916–1918).

Type species: *Pupa acarus* Benson, 1856

*Gastrocopta huttoniana* (Benson, 1849)

Distribution: India: Western Ghats, Himachal Pradesh, Kashmir, Maharashtra (Ramakrishna et al. 2010, Raheem et al. 2014).

Nepal: Annapurna Conservation Area (Kuznetsov and Schileyko 1997).

Superfamily: Enoidea Woodward, 1903

Family: Enidae Woodward, 1903

Subfamily: Pseudonapaeinae Schileyko, 1978

Genus: *Pupinidius* Möllendorff, 1901

Distribution: W China; Nepal (Schileyko 1998, Wu and Zheng 2009).

Type species: *Buliminus pupinidius* Möllendorff, 1901

*Pupinidius himalayanus* Kuznetsov & Schileyko, 1999

Distribution: Endemic to Nepal.

Nepal: Mustang District, Tukuche to Muktinath trekking route (Kuznetsov and Schileyko 1999).

*Pupinidius siniayevi* Kuznetsov & Schileyko, 1999

Distribution: Endemic to Nepal.

Nepal: Mustang District-Tukuche to Muktinath trekking route (Kuznetsov and Schileyko 1999).

*Pupinidius tukuchensis* Kuznetsov & Schileyko, 1997

Distribution: Endemic to Nepal.

Nepal: Mustang District-Tukuche (Kuznetsov and Schileyko 1997).

Genus: *Laevozebrinus* Lindholm, 1925

Distribution: Afghanistan; Iran; mountain regions of C Asia; N Pakistan and adjacent territories of India (Schileyko 1998).

Type species: *Buliminus urgutensis* Kobelt, 1902

*Laevozebrinus mustangensis* Kuznetsov & Schileyko, 1997

Distribution: Endemic to Nepal.

Nepal: Mustang District-Tukuche to Muktinath trekking route (Kuznetsov and Schileyko 1997).

*Laevozebrinus nepalensis* Schileyko & Frank, 1994

Distribution: Endemic to Nepal.

Nepal: Annapurna Conservation Area and hills surrounding Kathmandu District (Schileyko and Frank 1994).

Subspecies: *nepalensis* Schileyko & Frank, 1994

Distribution: Mustang District-Khobang, Tukuche, Marpha, Jomsom (Kuznetsov and Schileyko 1997).

Subspecies: *myagdiensis* Kuznetsov & Schileyko, 1997

Distribution: Myagdi District-Sukebagar, Titre, Dana (Kuznetsov and Schileyko 1997).

Genus: *Mirus* Albers, 1850

Distribution: India; Sri Lanka; Myanmar; E Asia; Japan (Kuznetsov and Schileyko 1997, Schileyko 1998, Raheem and Naggs 2006).

Type species: *Bulimus cantorii* Philippi, 1844^20^

Mirus (?) nilagiricus (L. Pfeiffer, 1846)^21^

Distribution: India: Western Ghats, Tamil Nadu-Nilgiris, Arunachal Pradesh-Dafla Hill, Meghalaya-Khasi Hills; Myanmar (Gude 1914, Ramakrishna et al. 2010, Raheem et al. 2014).

Nepal: Solukhumbu District-Khari Khola (Kuznetsov and Schileyko 1997).

Genus: *Nepaliena* Schileyko & Frank, 1994

Distribution: Endemic to Nepal (Kuznetsov and Schileyko 1997).

Type species: *Bulimus ceratinus* Benson, 1849

*Nepaliena ceratina* (Benson, 1849)

Distribution: Endemic to Nepal.

Nepal: Kathmandu and Myagdi Districts, Annapurna Conservation Area (Schileyko and Frank 1994, Kuznetsov and Schileyko 1997).

Genus: *Subzebrinus* Westerlund, 1887

Distribution: SE Kazakhstan and adjacent territories of China; India; Japan; Nepal (Gude 1914, Schileyko 1998, Raheem et al. 2010).

Type species: *Buliminus labiellus* Martens, 1881

*Subzebrinus rufistrigatus* (Reeve, 1849)

Distribution: India: Kashmir between Jamuna and Sutlej River, Jhelum Valley (Gude 1914)

New species record for Nepal: Mugu District-Rogumba.

Family: Cerastidae Wenz, 1923

Genus: *Darwininitium* Budha & Mordan, 2012^22^

Distribution: Endemic to Nepal.

Type species: *Darwininitium shiwalikianum* Budha & Mordan, 2012

*Darwininitium shiwalikianum* Budha & Mordan, 2012

Distribution: Endemic to Nepal.

Nepal: Shiwalik range of C Nepal, Chitwan National Park and Makwanpur District-Taubas, Bhaise (Budha et al. 2012).

Superfamily: Clausilioidea J.E. Gray, 1855

Family: Clausiliidae J.E. Gray, 1855

Subfamily: Phaedusinae A.J. Wagner, 1922

Genus: *Cylindrophaedusa* O. Boettger, 1877^23^

Distribution: Pakistan; India; Nepal; Bhutan; Myanmar (Nordsieck 2002).

Type species: *Clausilia cylindrica* L. Pfeiffer, 1846

Subgenus: *Cylindrophaedusa* O. Boettger, 1877

Distribution: India: Punjab, W Bengal (Nordsieck 1973, 2002).

Type species: *Clausilia cylindrica* L. Pfeiffer, 1846

Cylindrophaedusa (Cylindrophaedusa) cylindrica (L. Pfeiffer, 1846)

Distribution: India: Punjab-Muree, W Bengal-Darjeeling (Nordsieck 1973, 2002).

New species record for Nepal: Dadeldhura District.

Subgenus: *Montiphaedusa* Nordsieck, 2002

Distribution: N Pakistan; Nepal; NE India; Bhutan; Myanmar (Nordsieck 2002).

Type species: *Clausilia ioes* Benson, 1852

Cylindrophaedusa (Montiphaedusa) ioes (Benson, 1852)

Distribution: N Pakistan; Nepal; NE India; Bhutan; Myanmar (Nordsieck 2002).

Subspecies: *jiriensis* (Nordsieck, 1973)

Distribution: Endemic to Nepal.

Nepal: Dolakha District-Jiri (Nordsieck 1973).

Cylindrophaedusa (Montiphaedusa) kathmandica (Nordsieck, 1973)

Distribution: Endemic to Nepal.

Nepal: Hills surrounding Kathmandu Valley (Nordsieck 1973).

New distribution records from Nepal: Lalitpur District-Phulchowki Hill, Shivapuri-Nagarjun and Langtang National Parks.

Cylindrophaedusa (Montiphaedusa) martensiana (Nordsieck, 1973)

Distribution: Endemic to Nepal.

Nepal: Lamjung, Myagdi and Mustang Districts (Nordsieck 1973).

Subspecies: *martensiana* (Nordsieck, 1973)

Distribution: Myagdi and Mustang Districts-Dhorpatan, Thakkhola, Lete, Gorepani (Nordsieck 1973).

Subspecies: *dhaulagirica* (Nordsieck, 1973)

Distribution: Lamjung District-Jaljala, Myagdi Khola, Muri (Nordsieck 1973).

Superfamily: Achatinoidea Swainson, 1840

Family: Achatinidae Swainson, 1840

Genus: *Lissachatina* Bequaert, 1950^24^

Distribution: Originally from E Africa but now globally distributed in tropical to warm temperate areas, i.e. W Africa; N and S America; S and SE Asia; China; Japan; Caribbean countries; Oceania (Tillier et al. 1993, Raut and Barker 2002, EPPO 2013).

Type species: *Achatina fulica* Bowdich, 1822

*Lissachatina fulica* (Bowdich, 1822)

Distribution: See distribution of *Lissachatina*.

Nepal: Probably introduced into Nepal in the 1930s-40s (Raut 1999). It is now established as a pest in all districts of Tarai and the inner valleys causing significant damage to crops. It has spread into the mid hill districts: Kaski, Baglung, Makwanpur, Chitwan, Myagdi, Tanahun, Dhading, Palpa, Gulmi, Syangjha (Budha and Naggs 2008).

New distribution records from Nepal: Dang, Surkhet, Banke, Bardia, Kailali and Kanchanpur Districts.

Family: Ferussaciidae Bourguignat, 1883

Genus: *Cecilioides* Férussac, 1814^25^

Distribution: Europe; Africa; S Asia; Philippines; Oceania; American tropics (Thompson 2011).

Type species: *Buccinum acicula* O.F. Müller, 1774

Cecilioides cf. minuta Mousson, 1874^26^

Distribution: Drift debris of the Euphrates (type locality), Sarus River near Adana, SE Asia Minor (Pilsbry and Tryon 1908–1909).

New species record for Nepal: Baitadi District, Far W Nepal.

Family: Subulinidae P. Fischer & Crosse, 1877

Subfamily: Subulininae P. Fischer and Crosse, 1877^27^

Genus: *Allopeas* H.B. Baker, 1935^28^

Distribution: Tropical, subtropical, and many temperate regions of Africa, S and SE Asia (Schileyko 1999, Thompson 2011).

Type species: *Bulimus gracilis* Hutton, 1834 (= *Allopeas gracile* (Hutton, 1834))

*Allopeas clavulinum* (Potiez & Michaud, 1838)^29^

Distribution: Bourbon Island (type locality), other islands of the Indian Ocean; Japan (Pilsbry 1946).

New species records for Nepal: Kathmandu, Kaski and Kailali Districts.

*Allopeas gracile* (Hutton, 1834)

Distribution: Tropics of both hemispheres, abundant in cultivated districts, perhaps the most widely ranging of all land snails (Pilsbry 1946).

New species records for Nepal: Chitwan and Dhading Districts.

Genus: *Curvella* Chaper, 1885

Distribution: S Africa; India; China; SE Asia (Gude 1914, Schileyko 1999).

Type species: *Curvella sulcata* Chaper, 1885

*Curvella sikkimensis* Gude, 1914

Distribution: India: W Bengal-Darjeeling, Sikkim (Gude 1914).

New species record for Nepal: Ilam District-Maipokhari.

Genus: *Paropeas* Pilsbry, 1906

Distribution: Widespread in the tropical Indo-Pacific regions (Naggs 1994).

Type species: *Bulimus acutissimum* Mousson, 1857

*Paropeas achatinaceum* (L.Pfeiffer, 1846)

Distribution: Widespread in disturbed habitats in tropical Indo-Pacific regions (Naggs 1994).

Nepal: Shivapuri-Nagarjun National Park-Nagarjun Forest (Khanal and Budha 2013).

New distribution record from Nepal: Ramechhap District.

Subfamily: Opeatinae Thiele, 1931

Genus: *Opeas* Albers, 1850^30^

Distribution: Worldwide in tropical, subtropical and many temperate regions (Schileyko 1999, Thompson 2011).

Type species: *Helix goodallii* Miller, 1822

*Opeas* sp.

Nepal: Morang District (Subba and Ghosh 2008).

Subfamily: Glessulinae Godwin-Austen, 1920^31^

Genus: *Bacillum* Theobald, 1870^32^

Distribution: NE India: W Bengal-Darjeeling, Sikkim, Assam-North Cachar, Meghalaya-Khasi Hill, Nagaland-Naga Hills (Gude 1914, Ramakrishna et al. 2010).

Type species: *Achatina cassiaca* Reeve, 1849

*Bacillum* sp.^33^

Nepal: Ilam and Panchthar District (Subba and Ghosh 2008).

Genus: *Glessula* Martens, 1860

Distribution: India; Sri Lanka; Thailand; Malaysia; Vietnam (Gude 1914, Godwin-Austen 1920, Schileyko 2011).

Nepal: Kathmandu District (Kuznetsov 1996, Schileyko and Kuznetsov 1996).

Type species: *Achatina ceylanica* L. Pfeiffer, 1845^34^

*Glessula orobia* (Benson, 1860)

Distribution: India: W Bengal-Darjeeling (Gude 1914, Ramakrishna et al. 2010).

New species record for Nepal: Ilam District-Maipokhari.

*Glessula subjerdoni* Beddome, 1906^35^

Distribution: S India: Western Ghats, Andhra Pradesh-Golconda Hill, Orissa-Jaypore (Gude 1914, Ramakrishna et al. 2010, Raheem et al. 2014).

Nepal: Kathmandu District-Nagarjun Forest (Kuznetsov 1996).

Genus: *Rishetia* Godwin-Austen, 1920

Distribution: India; Sri Lanka; Nepal; Myanmar; W Bhutan (Godwin-Austen 1920)^36^.

Type species: Glessula (Rishetia) longispira Godwin-Austen, 1920

*Rishetia hastula* (Benson, 1860)

Distribution: India: W Bengal-Darjeeling (Gude 1914, Godwin-Austen 1920).

New species record for Nepal: Chitwan National Park.

*Rishetia tenuispira* (Benson, 1836)^37^

Distribution: India: Western Ghats, W Bengal, Sikkim, Mizoram, Arunachal Pradesh, Maharastra; Myanmar; Bangladesh (Pilsbry and Tryon 1908-1909, Gude 1914, Ramakrishna et al. 2010, Raheem et al. 2014).

Nepal: Shivapuri-Nagarjun National Park-Nagarjun Forest, Balaju (Schileyko and Kuznetsov 1996).

New distribution record from Nepal: Lalitpur District-Phulchowki Hill.

Superfamily: Streptaxoidea J.E. Gray, 1860

Family: Streptaxidae J.E. Gray, 1860

Genus: *Gulella* L. Pfeiffer, 1856

Distribution: Africa; Indo-Pacific (Bruggen 2006, Cole and Herbert 2009, Herbert and Rowson 2011, Rowson et al. 2011).

Type species: *Pupa menkeana* L. Pfeiffer, 1853

*Gulella bicolor* (Hutton, 1834)^38^

Distribution: Sri Lanka; throughout India; Myanmar (Blanford and Godwin-Austen 1908); Brazil (Pilsbry 1926, Simone 2013); SE China (Yen 1939); Cuba (Sarasúa 1944, Maceira et al. 2013); Caribbean Islands (Schalie 1948); Philippines; Indonesia (Benthem Jutting 1950); Andaman and Nicobar Islands; Malaysia; Singapore (Benthem Jutting 1961); Kenya (Clench 1964); Venezuela; French Guiana (Tillier 1980); Japan (Azuma 1982); Australia (Stanisic 1981); N America (Dundee and Baerwold 1984); Oman (Mordan 1988); Bel Air area of North Mahé; Seychelles (Naggs 1989); Jamaica (Rosenberg and Muratov 2006); Vietnam (Schileyko 2011).

New species record for Nepal: Chitwan District.

Family: Diapheridae Panha & Naggs, 2010

Subfamily: Enneinae Bourguignat, 1883

Genus: *Sinoennea* Kobelt, 1904

Distribution: Japan; China; Vietnam; Malaysia; Sumatra; India (Gude 1914).

Type species: *Pupa strophioides* Gredler, 1881

Subgenus: *Indoennea* Kobelt, 1904

Distribution: India; Malaysia; Sumatra (Schileyko 2000).

Type species: *Ennea blanfordiana* Godwin-Austen, 1872

Sinoennea (Indoennea) blanfordiana Godwin-Austen, 1872

Distribution: India: Assam-North Cachar (Ramakrishna et al. 2010).

New species record for Nepal: Lalitpur District-Phulchowki Hill.

Subgenus: *Sinoennea* Kobelt, 1904

Distribution: Foothills of Himalaya; S India; China; Malay Peninsula; Sumatra; Japan; S Korea (Schileyko 2000).

Type species: *Pupa strophioides* Gredler, 1881

Sinoennea (Sinoennea) stenopylis (Benson, 1860)

Distribution: NE India: Arunachal Pradesh, Sikkim, Assam, Manipur, Meghalaya, Nagaland (Ramakrishna et al. 2010).

Nepal: Solukhumbu District (Kuznetsov and Schileyko 1997).

Superfamily: Plectopyloidea Möllendorff, 1898

Family: Plectopylidae Möllendorff, 1898

Genus: *Endothyrella* Zilch, 1960^39^

Distribution: Nepal; NE India: Arunachal Pradesh, Assam, Nagaland, Meghalaya, Manipur, Mizoram, Sikkim (Ramakrishna et al. 2010).

Type species: *Helix plectosoma* Benson, 1836

*Endothyrella affinis* (Gude, 1897)^40^

Distribution: NE India: Arunachal Pradesh, Assam, Meghalaya-Khasi Hill, Mizoram (Gude 1914, Ramakrishna et al. 2010).

Nepal: Kathmandu District-Swoyambhunath Temple Forest (Kuznetsov and Schileyko 1997).

*Endothyrella minor* (Godwin-Austen, 1879)

Distribution: India: Manipur, Meghalaya, Nagaland, Sikkim, W Bengal (Gude 1914, Ramakrishna et al. 2010).

New species records for Nepal: Lalitpur District-Phulchowki Hill, Shivapuri-Nagarjun National Park-Chisapani, Baghdwar, Langtang National Park-Golphubhanjyang.

Superfamily: Gastrodontoidea Tryon, 1866

Family: Chronidae Thiele, 1931

Subfamily: Kaliellinae Thiele, 1931

Genus: *Kaliella* W.T. Blanford, 1863

Distribution: Indo-Malayan (Blanford and Godwin-Austen 1908).

Type species: *Helix barrakporensis* L. Pfeiffer, 1853^41^

*Kaliella barrakporensis* (L. Pfeiffer, 1853)

Distribution: India; Sri Lanka; Pakistan; Madagascar; Myanmar; Tropical E Africa and Eastern S Africa (Blanford and Godwin-Austen 1908, Herbert and Kilburn 2004, Verdcourt 2006, Ramakrishna et al. 2010), hot-house alien in Britain (Preece and Naggs 2014).

Nepal: Annapurna Conservation Area (Kuznetsov and Schileyko 1977).

New distribution records from Nepal: Shivapuri-Nagarjun National Park, Lalitpur District-Phulchowki Hill, Kathmandu District-Champadevi Hill, Kirtipur.

*Kaliella dikrangensis* Godwin-Austen, 1883

Distribution: India: Arunachal Pradesh (Blanford and Godwin-Austen 1908, Ramakrishna et al. 2010).

Nepal: Shivapuri-Nagarjun National Park (Khanal and Budha 2013).

*Kaliella fastigiata* (Hutton, 1838)

Distribution: India: Himachal Pradesh, Uttarakhand, W Bengal, Arunachal Pradesh, Nagaland; Madagascar; Myanmar (Blanford and Godwin-Austen 1908, Ramakrishna et al. 2010).

Nepal: Kathmandu District-Champadevi Hill (Khanal and Budha 2013).

New distribution record from Nepal: Lalitpur District-Phulchowki Hill.

*Kaliella nana* (Hutton, 1838)

Distribution: India: Uttarakhand, Himachal Pradesh, W Bengal (Blanford and Godwin-Austen 1908, Ramakrishna et al. 2010).

Nepal: Annapurna Conservation Area (Kuznetsov and Schileyko 1997), Shivapuri-Nagarjun National Park (Khanal and Budha 2013).

New distribution records from Nepal: Lalitpur District-Phulchowki Hill and Kathmandu District-Champadevi Hill.

*Kaliella nongsteinensis* Godwin-Austen, 1883

Distribution: India: Meghalaya-Khasi Hill (Blanford and Godwin-Austen 1908, Ramakrishna et al. 2010).

Nepal: Solukhumbu District (Kuznetsov and Schileyko 1997).

Family: Euconulidae H.B. Baker, 1928

Subfamily: Euconulinae H.B. Baker, 1928

Genus: *Euconulus* Reinhardt, 1883

Distribution: Holarctic (Roth and Sadeghian 2006).

Type species: *Helix fulva* O.F. Müller, 1774

*Euconulus fulvus* (O.F. Müller, 1774)^42^

Distribution: Holarctic (Roth and Sadeghian 2006).

Nepal: Hills surrounding Kathmandu Valley (Kuznetsov and Schileyko 1997).

New distribution records from Nepal: Langtang National Park and Mustang District.

Family: Pristilomatidae Cockerell, 1891

Genus: *Hawaiia* Gude, 1911^43^

Distribution: N America from Alaska and Maine to Florida and south to Costa Rica, Cuba, Hispaniola, Jamaica, Puerto Rico, and the Virgin Islands; Europe; Japan; Australia (Kerney and Cameron 1979, Rosenberg and Muratov 2006, Sasaki 2008, Thompson 2011).

Type species: *Helix kawaiensis* Reeve, 1854 (= *Helix minuscula* Binney, 1841)

*Hawaiia* sp.

Nepal: Annapurna Conservation Area (Kuznetsov and Schileyko 1997).

Superfamily: Helicarionoidea Bourguignat, 1877

Family: Helicarionidae Bourguignat, 1877

Subfamily: Durgelinae Godwin-Austen, 1888

Genus: *Durgella* W.T. Blanford, 1863

Distribution: India: Arunachal Pradesh, Assam, Andhra Pradesh, Orissa, Meghalaya, Manipur, Sikkim, W Bengal; Myanmar (Blanford and Godwin-Austen 1908, Ramakrishna et al. 2010).

Type species: *Helix levicula* Benson, 1859

*Durgella* sp.

New species records for Nepal: Chitwan National Park; Kathmandu and Pokhara Districts.

Genus: *Sitala* H. Adams, 1865

Distribution: India; Sri Lanka; Andaman Islands; SE Asia (Schileyko 2002).

Type species: *Helix infula* Benson, 1848

*Sitala rimicola* (Benson, 1859)

Distribution: India: Uttarakhand, W Bengal, Sikkim, Assam, Meghalaya, Nagaland (Ramakrishna et al. 2010).

Nepal: Mustang District (Kuznetsov and Schileyko 1997).

New distribution records from Nepal: Dadeldhura, Kathmandu, Rasuwa and Mustang Districts.

Genus: *Cryptaustenia* Cockerell, 1891^44^

Distribution: India; Nepal; Bhutan; Myanmar; Thailand (Schileyko 2003).

Type species: *Vitrina planospira* Benson, 1859 (= *Vitrina succinea* Reeve, 1862)

Cryptaustenia cf. globosa (Godwin-Austen, 1876)

Distribution: India: Arunachal Pradesh (Ramakrishna et al. 2010).

Nepal: Kathmandu District, Annapurna Conservation Area (Kuznetsov and Schileyko 1997).

*Cryptaustenia ovata* (H.F. Blanford, 1871)

Distribution: India: W Bengal (Blanford and Godwin-Austen 1908).

Nepal: Kathmandu, Panchthar, Taplejung, Morang and Terhathum Districts (Subba and Ghosh 2008), Annapurna Conservation Area (Kuznetsov and Schileyko 1997). Shivapuri-Nagarjun National Park-Nagarjun Forest (Khanal and Budha 2013).

Genus: *Girasia* J.E. Gray, 1855

Distribution: India: Assam, Arunachal Pradesh, Himachal Pradesh, Meghalaya, Mizoram, Manipur, Nagaland, Sikkim; Myanmar (Blanford and Godwin-Austen 1908, Ramakrishna et al. 2010).

Type species: *Girasia hookeri* J.E. Gray, 1855

*Girasia* sp.

New species record for Nepal: Langtang National Park.

Family: Ariophantidae Godwin-Austen, 1888

Subfamily: Macrochlamydinae Godwin-Austen, 1888

Genus: *Macrochlamys* Benson in Godwin-Austen, 1883^45^

Distribution: S and SE Asia (Blanford and Godwin-Austen 1908).

Type species: *Macrochlamys indica* Benson in Godwin-Austen, 1883

*Macrochlamys indica* Benson in Godwin-Austen, 1883

Distribution: India; Andaman Islands; Bangladesh; Sri Lanka (Ramakrishna et al. 2010).

Nepal: Ilam, Sunsari, Dharan, Kathmandu, Lalitpur, Gulmi, Kaski Districts (Subba and Ghosh 2001).

New distribution records from Nepal: Dadeldhura, Baitadi, and Kanchanpur Districts.

*Macrochlamys lata* Godwin-Austen, 1888

Distribution: India: Meghalaya (Ramakrishna et al. 2010).

Nepal: Annapurna Conservation Area (Kuznetsov and Schileyko 1997).

*Macrochlamy longicauda* Godwin-Austen, 1883

Distribution: India: Meghalaya (Ramakrishna et al. 2010).

Nepal: Kathmandu District, Annapurna Conservation Area (Kuznetsov and Schileyko 1997).

New distribution records from Nepal: Shivapuri-Nagarjun and Langtang National Parks.

*Macrochlamys lubrica* (Benson, 1852)

Distribution: India: W Bengal-Darjeeling, Sikkim, Meghalaya (Blanford and Godwin-Austen 1908, Ramakrishna et al. 2010).

Nepal: Mid hills of several districts of E Nepal (Subba and Ghosh 2008).

*Macrochlamys nuda* (L. Pfeiffer, 1852)

Distribution: NW India: Himachal Pradesh-Simla, Uttarakhand-Kumaon (Blanford and Godwin-Austen 1908).

Nepal: Annapurna Conservation Area (Schileyko and Kuznetsov 1996, 1998b).

*Macrochlamys patane* (Benson, 1859)

Distribution: NE India: W Bengal-Darjeeling, Sikkim (Blanford and Godwin-Austen 1908).

Nepal: Kathmandu District (Schileyko and Kuznetsov 1996).

*Macrochlamys perpaula* (Benson, 1859)

Distribution: India: Bihar, Jharkhand, Sikkim, W Bengal-Darjeeling (Ramakrishna et al. 2010).

Nepal: Shivapuri-Nagarjun National Park-Nagarjun Forest (Khanal and Budha 2013).

*Macrochlamys sathilaensis* Godwin-Austen, 1907

Distribution: NE India: Sikkim-Richila Peak; Bhutan (Blanford and Godwin-Austen 1908).

Nepal: Annapurna Conservation Area, Solukhumbu District (Kuznetsov and Schileyko 1997).

*Macrochlamys sequax* (Benson, 1859)

Distribution: India: W Bengal-Darjeeling (Blanford and Godwin-Austen 1908).

Nepal: Annapurna Conservation Area (Kuznetsov and Schileyko 1997).

*Macrochlamys sequius* Godwin-Austen, 1907

Distribution: India: W Bengal-Darjeeling (Blanford and Godwin-Austen 1908).

Nepal: Annapurna Conservation Area (Kuznetsov and Schileyko 1997).

*Macrochlamys subjecta* (Benson, 1852)

Distribution: India: Jharkhand-Rajmahal Hills, Orrissa-Cuttak (Blanford and Godwin-Austen 1908).

Nepal: Annapurna Conservation Area (Kuznetsov and Schileyko 1997).

New distribution records from Nepal: Widely distributed in W Tarai to the mid hills of C Nepal.

*Macrochlamys tugurium* (Benson, 1852)^46^

Distribution: India: Manipur, Sikkim, W Bengal-Darjeeling (Ramakrishna et al. 2010).

Nepal: Kathmandu District (Kiauta and Butot 1972).

New distribution record from Nepal: Khaptad National Park.

Genus: *Euaustenia* Cockerell, 1891^47^

Distribution: Afghanistan; Pakistan; NW and NE India: Uttarakhand, Sikkim (Blanford and Godwin-Austen 1908).

Type species: *Vitrina scutella* Benson, 1859 (= *Vitrina monticola* L. Pfeiffer, 1849^48^)

*Euaustenia cassida* (Hutton, 1838)

Distribution: NW India: Himachal Pradesh, Kashmir, Uttarakhand-Kumaon (Blanford and Godwin-Austen 1908, Ramakrishna et al. 2010).

New species records for Nepal: Baitadi, Darchula, and Dadeldhura Districts.

*Euaustenia monticola* (L. Pfeiffer, 1849)

Distribution: NW India: Kashmir, Uttarakhand-Nainital (Ramakrishna et al. 2010).

Nepal: Kathmandu District (Schileyko and Frank 1994, Kuznetsov 1996), Annapurna Conservation Area (Kuznetsov and Schileyko 1997).

New distribution records from Nepal: Shivapuri-Nagarjun and Langtang National Parks.

Genus: *Bensonies* H.B. Baker, 1938

Distribution: Afghanistan; Pakistan; India: Uttarakhand, Sikkim (Blanford and Godwin-Austen 1908).

Type species: *Nanina monticola* Hutton, 1838

*Bensonies convexa* (Reeve, 1852)

Distribution: India: Himachal Pradesh, Uttarakhand (Blanford and Godwin-Austen 1908).

Nepal: Annapurna Conservation Area (Kuznetsov and Schileyko 1997).

New distribution records from Nepal: Lalitpur District-Phulchowki Hill, Kathmandu District- Champadevi Hill, Shivapuri-Nagarjun and Langtang National Parks.

*Bensonies jacquemonti* (Martens, 1869)

Distribution: Pakistan: Murree; NW India: Himachal Pradesh, Kashmir, Punjab, Uttarakhand (Blanford and Godwin-Austen 1908, Ramakrishna et al. 2010).

New species record for Nepal: Baitadi District.

*Bensonies monticola* (Hutton, 1838)

Distribution: NW India: Kashmir, Punjab, Uttarakhand (Blanford and Godwin-Austen 1908, Ramakrishna et al. 2010).

New species record for Nepal: Khaptad National Park.

*Bensonies nepalensis* (W.T. Blanford, 1904)^49^

Distribution: Endemic to Nepal, where it is common in Kathmandu Valley (Blanford 1904, Blanford and Godwin-Austen 1908).

New distribution records from Nepal: Lalitpur, Kavre, Chitwan, Kaski, Gulmi, Syangjha, Parbat, and Myagdi Districts.

*Bensonies theobaldiana* (Godwin-Austen, 1888)

Distribution: NW India: Himachal Pradesh-Simla, Uttarakhand (Blanford and Godwin-Austen 1908, Ramakrishna et al. 2010).

New species record for Nepal: Khaptad National Park.

Genus: *Himalodiscus* Kuznetsov, 1996

Distribution: Endemic to Nepal.

Nepal: C and W Nepal.

Type species: *Himalodiscus aculeatus* Kuznetsov, 1996

*Himalodiscus aculeatus* Kuznetsov, 1996^50^

Distribution: Endemic to Nepal.

Nepal: Lalitpur District-Phulchowki Hill (Kuznetsov 1996).

New distribution record from Nepal: Shivapuri-Nagarjun National Park.

*Himalodiscus echinatus* Schileyko & Kuznetsov, 1998

Distribution: Endemic to Nepal.

Nepal: Annapurna Conservation Area. Only reported from the type locality Lete (Schileyko and Kuznetsov 1998b).

Genus: *Khasiella* Godwin-Austen, 1899

Distribution: E Himalaya from Nepal and India to Myanmar (Blanford and Godwin-Austen 1908).

Type species: *Helix vidua* Hanley & Theobald, 1875^51^

*Khasiella ornatissima* (Benson, 1859)

Distribution: India: W Bengal, Sikkim (Blanford and Godwin-Austen 1908), Uttar Pradesh (Ramakrishna et al. 2010).

Nepal: Lalitpur District-Phulchowki Hill (Kuznetsov 1996).

New distribution records from Nepal: Chitwan National Park, Chitwan and Nawalparasi Districts.

*Khasiella pansa* (Benson, 1856)^52^

Distribution: Myanmar: Ayeyarwady Valley, Sullivan Island, Mergui Archipelago (Blanford and Godwin-Austen 1908).

Nepal: Ilam, Jhapa, Morang, Sunsari, Dharan, Saptari, Udayapur, Kaski, Rupandehi and Kailali Districts (Subba and Ghosh 2001, Subba 2003, Surana et al. 2004).

Genus: *Oxytesta* Zilch, 1956

Distribution: E Himalaya from Nepal and NE India to Myanmar and Laos (Blanford and Godwin-Austen 1908).

Type species: *Helix oxytes* Benson, 1836

*Oxytesta blanfordi* (Theobald, 1859)

Distribution: India: W Bengal-Darjeeling, Sikkim (Blanford and Godwin-Austen 1908, Ramakrishna et al. 2010).

Nepal: Mustang (Kuznetsov and Schileyko 1997, Schileyko and Kuznetsov 1998b).

New distribution records from Nepal: Rasuwa and Parbat Districts.

*Oxytesta cycloplax* (Benson, 1852)

Distribution: India: Sikkim (Ramakrishna et al. 2010).

Nepal: Solukhumbu District (Kuznetsov and Schileyko 1997).

New distribution record from Nepal: Sankhuwasabha District.

*Oxytesta orobia* (Benson, 1848)^53^

Distribution: India: W Bengal-Darjeeling (Ramakrishna et al. 2010).

Nepal: Hills surrounding Kathmandu Valley (Schileyko and Frank 1994, Kuznetsov and Schileyko 1997).

New distribution records from Nepal: Shivapuri-Nagarjun and Langtang National Parks, Sankhuwasabha District.

*Oxytesta sylvicola* (W.T. Blanford, 1880)

Distribution: NE India: Assam-Burail range, North Cachar, Nagaland (Blanford and Godwin-Austen 1908, Ramakrishna et al. 2010).

Nepal: Ilam, Morang, Dharan, Udayapur, Kaski, Kathmandu, Lalitpur and Terhathum Districts (Subba and Ghosh 2001).

Genus: *Rotungia* Godwin-Austen, 1918

Distribution: India: Arunachal Pradesh-Abor Hill; Myanmar-Upper Rotung (Ramakrishna et al. 2010).

Type species: *Rotungia williamsoni* Godwin-Austen, 1918

*Rotungia williamsoni* Godwin-Austen, 1918

Distribution: India: Arunachal Pradesh-Abor Hill (Ramakrishna et al. 2010)

Nepal: Taplejung and Terhathum Districts (Subba and Ghosh 2008).

Genus: *Syama* Blanford & Godwin-Austen, 1908

Distribution: India (Blanford and Godwin-Austen 1908).

Type species: Nanina (Macrochlamys) prona Nevill, 1878.

*Syama prona* (Nevill, 1878)

Distribution: NW India: Himachal Pradesh, Uttarakhand (Blanford and Godwin-Austen 1908, Ramakrishna et al. 2010).

Nepal: Shivapuri-Nagarjun National Park-Nagarjun Forest (Khanal and Budha 2013).

Subspecies: *prona* (Nevill, 1878)

Distribution: Annapurna Conservation Area (Kuznetsov and Schileyko 1997, Schileyko and Kuznetsov 1998b).

Genus: *Rasama* Laidlaw, 1932^54^

Distribution: NE India; W Bhutan (Blanford and Godwin-Austen 1908).

Type species: *Macrochlamys kala* Godwin-Austen, 1883

*Rasama kala* (Godwin-Austen, 1883)

Distribution: India: Sikkim-Damsang Peak, Dalling Hills; W Bhutan (Blanford and Godwin-Austen 1908).

New species record for Nepal: Ilam District-Maipokhari.

Genus: *Taphrospira* W.T. Blanford, 1905

Distribution: India: Assam; Andaman Islands; Myanmar (Blanford and Godwin-Austen 1908).

Type species: *Helix convallata* Benson, 1856

*Taphrospira compluvialis* (W.T. Blanford, 1865)

Distribution: India: Assam; Andaman Islands; Myanmar (Blanford and Godwin-Austen 1908).

Nepal: Panchthar, Taplejung and Terhathum Districts (Subba and Ghosh 2008).

*Taphrospira convallata* (Benson, 1856)

Distribution: Myanmar (Blanford and Godwin-Austen 1908).

New species record for Nepal: Shivapuri-Nagarjun National Park.

Superfamily: Limacoidea Lamarck, 1801

Family: Limacidae Lamarck, 1801

Subfamily: Limacinae Lamarck, 1801

Distribution: W Palearctic region (Wiktor and Bössneck 2004).

Genus: *Limax* Linnaeus, 1758

Distribution: Palearctic region (Wiktor and Bössneck 2004).

Type species: *Limax maximus* Linnaeus, 1758

*Limax seticus* Wiktor & Bössneck, 2004

Distribution: This is the only *Limax* species recorded from the Himalaya (Wiktor and Bössneck 2004).

Nepal: Endemic to Nepal; probably the slug species with the highest elevation range (up to 5000 m) in the world. This species was reported only from Bajura District.

Genus: *Turcomilax* Simroth, 1901

Distribution: India and Nepal (Wiktor et al. 1999, Bössneck 2006).

Type species: Gigantomilax (Turcomilax) nanus Simroth, 1901

Subgenus: *Kasperia* Godwin-Austen, 1914^55^

Distribution: India: Kashmir (Godwin-Austen, 1914).

Type species: Limax (Kasperia) mayae Godwin-Austen, 1914 (= *Limax turkestanus* Simroth, 1898)

Turcomilax (Kasperia) oli Wiktor, Naggs & Gupta, 1999^56^

Distribution: India: Kumaun Himalaya (Wiktor et al. 1999).

Nepal: Darchula District (Bössneck 2006).

Family: Agriolimacidae Wagner, 1935

Subfamily: Agriolimacinae Wagner, 1935

Genus: *Deroceras* Rafinesque, 1820

Distribution: Holarctic. From Sahara to NE America and S Asia (Wiktor et al. 2000, Thompson 2011).

Type species: *Limax laevis* O.F. Müller, 1774

*Deroceras laeve* (O.F. Müller, 1774)

Distribution: Holarctic. From Sahara to NE America. It has been introduced worldwide (Wiktor et al. 2000).

Nepal: Kathmandu, Taplejung and Panchthar Districts (Bössneck 2006).

New distribution record from Nepal: Lalitpur District.

Superfamily: Arionoidea J.E. Gray, 1840

Family: Anadenidae Pilsbry, 1948

Genus: *Anadenus* Heynemann, 1863

Distribution: S China; southern slopes of the Himalaya from Pakistan eastward to Sikkim (Wiktor 2001a).

Type species: *Anadenus giganteus* Heynemann, 1863 [Currently replaced by: *Limax altivagus* Theobald, 1862]^57^

*Anadenus altivagus* (Theobald, 1862)^58^

Distribution: Southern slopes of the Himalaya from Rawalpindi in the west of N Pakistan through Kashmir and Nepal to Sikkim in NE India (Wiktor 2001a).

Nepal: Bajura, Darchula, Humla and Rasuwa Districts (Bössneck 2006).

New distribution records from Nepal: Langtang National Park-Dhunche-Gosainkund-Chisapani trekking route.

*Anadenus nepalensis* Wiktor, 2001

Distribution: Endemic to Nepal.

Nepal: Hills of Darchula, Dolpa, Humla, Jumla, Lamjung, Kaski, Palpa and Kathmandu Districts (Wiktor 2001a, Bössneck 2006).

Subgenus: *Sagarmathia* Kuzminykh & Schileyko, 2005

Distribution: Endemic to Nepal (Kuzminykh and Schileyko 2005).

Type species: Anadenus (Sagarmathia) kuznetsovi Kuzminykh & Schileyko, 2005

Anadenus (Sagarmathia) kuznetsovi Kuzminykh & Schileyko, 2005

Distribution: Endemic to Nepal.

Nepal: Only reported from the type locality, Phuiyan Khola, Solukhumbu District (Kuzminykh and Schileyko 2005).

Family: Philomycidae J.E. Gray, 1847

Genus: *Meghimatium* van Hasselt, 1823

Distribution: Russia; China; Korea; Japan; Borneo; Sumatra; Java; Celebes; Philippines (Wiktor and Jurkowska 2007).

Type species: *Meghimatium striatum* van Hasselt, 1823

Meghimatium cf. pictum (Stoliczka, 1873)^59^

Distribution: China; India (Wiktor et al. 2000).

Nepal: Chitwan National Park (Bössneck 2006).

Superfamily: Helicoidea Rafinesque, 1815

Family: Bradybaenidae Pilsbry, 1934

Subfamily: Bradybaeninae Pilsbry, 1934

Genus: *Bradybaena* Beck, 1837

Distribution: India; Bangladesh; Nepal; China; Myanmar; Thailand; Laos; Vietnam; Cambodia; Indonesia; Malaysia; Singapore (Hoong 1995, Panha 1995–1996, Wu 2002, 2004, Kuznetsov and Schileyko 1997, Ramakrishna et al. 2010, Schileyko 2011); the type species, *Bradybaena similaris* is widely introduced in other regions (Carvalho et al. 2008).

Type species: *Helix similaris* Férussac, 1821

*Bradybaena radicicola* (Benson, 1848)

Distribution: NW to NE India: Himachal Pradesh, Uttarakhand, Sikkim (Ramakrishna et al. 2010).

Nepal: Annapurna Conservation Area-Kokhethanti, Lete Khola (Kuznetsov and Schileyko 1997, Schileyko and Kuznetsov 1998a, 1998b).

*Bradybaena ? thakkholensis* Schileyko & Kuznetsov, 1998a^60^

Distribution: Endemic to Nepal.

Nepal: Annapurna Conservation Area. Only known from Thakkhola, the type locality (Schileyko and Kuznetsov 1998a).

Genus: *Plectotropis* Martens, 1860

Ditribution: India; China; Japan; Sumatra (Schileyko 2004, Ramakrishna et al. 2010).

Type species: *Helix elegantissima* L. Pfeiffer, 1849

*Plectotropis tapeina* (Benson, 1836)^61^

Distribution: India; Bangladesh; Myanmar (Ramakrishna et al. 2010).

Nepal: Ilam and Panchthar Districts (Subba and Ghosh 2008).

Family: Camaenidae Pilsbry, 1895

Subfamily: Camaeninae Pilsbry, 1895

Genus *Landouria* Godwin-Austen, 1918

Distribution: Sri Lanka; NE India; Nepal; Indonesia; Philippines (Schileyko and Kuznetsov 1998a).

Type species: *Helix huttonii* L. Pfeiffer, 1842^62^

*Landouria aborensis* Godwin-Austen, 1918

Distribution: India: Arunachal Pradesh-Abor Hill (Ramakrishna et al. 2010).

Nepal: Dolakha, Lalitpur, Ramechhap and Solukhumbu Districts (Kuznetsov and Schileyko 1997, Schileyko and Kuznetsov 1998a).

*Landouria coeni* (Preston, 1914)^63^

Distribution: India: Nagaland (Gude 1914).

Nepal: Solukhumbu District (Schileyko and Kuznetsov 1998a).

*Landouria dhaulagirica* Schileyko & Kuznetsov, 1998a

Distribution: Endemic to Nepal.

Nepal: Annapurna Conservation Area-Larjung, Kokhethanti, Kalopani (Schileyko and Kuznetsov 1998a).

*Landouria huttonii* (L. Pfeiffer, 1842)

Distribution: India: Himachal Pradesh, Uttarakhand, W Bengal, Assam, Nagaland (Ramakrishna et al. 2010).

Nepal: Kaski and Myagdi Districts (Kuznetsov and Schileyko 1997, Schileyko and Kuznetsov 1998a).

*Landouria rhododendronis* Schileyko & Kuznetsov, 1998a

Distribution: Endemic to Nepal.

Nepal: Annapurna Conservation Area-Gorepani, Parbat District (Schileyko and Kuznetsov 1998a).

*Landouria savadiensis* (Nevill, 1877)

Distribution: Myanmar: Sawady (Nevill, 1877).

Nepal: Shivapuri-Nagarjun National Park-Nagarjun Forest, Tare-Bhir (Schileyko and Kuznetsov 1998a).

Genus: *Ganesella* W.T. Blanford, 1863

Distribution: India; Myanmar; Thailand; Cambodia (Ramakrishna et al. 2010).

Type species: *Helix capitium* Benson, 1848

*Ganesella* sp.

Nepal: Shivapuri-Nagarjun National Park (Khanal and Budha 2013).

New distribution record from Nepal: Lalitpur District-Phulchowki Hill.

Notes1Eight subgenera of *Cyclophorus* were recognized by Kobelt (1902), one of which, the African *Maizania* Bourguignat, 1889, was elevated to family level Maizaniidae by Tielecke (1940) (see Bouchet and Rocroi 2005: 248). Gude (1921) mentioned only five subgenera viz.: *Glossostylus* Kobelt and Möllendorff, 1897 (S and SE Asia), *Litostylus* Kobelt & Möllendorff, 1897 (S and SE Asia), *Salpingophorus* Kobelt and Möllendorff, 1897 (S and SE Asia), *Cyclophorus* Montfort, 1810 (S and SE Asia) and *Cyclohelix* Mörch, 1852 (Andaman and Nicobar Islands). Wenz (1939: 458–460) replaced *Salpingophorus* by *Annularia* Schumacher, 1817 and *Cyclohelix* by *Otopoma* Gray, 1850, while Egorov (2006) replaced *Litostylus* by *Kobeltostylus* Egorov, 2006. In this list we follow Gude (1921) but with the adapted names proposed by Wenz (1939) and Egorov (2006). Note that Egorov and Greke (2007) also recognized five subgenera, but they regarded *Salpingophorus* as a junior synonym of *Cyclophorus*, while maintaining *Cricophorus* Kobelt and Möllendorff, 1897 as separate subgenus, next to *Cyclophorus*, *Glossostylus*, *Cyclohelix* and *Kobeltostylus*.2Allozyme data of *Cyclophorus fulguratus* populations in Thailand suggest that this is a species complex (Prasankok et al. 2009). It remains to be investigated how the Nepalese populations fit into this picture.3The name *Litostylus* Kobelt & Möllendorff, 1897 is a junior homonym of *Litostylus* Faust, 1893 (= Insecta, Coleoptera, Curculionidae). Egorov (2006) therefore replaced the molluscan name by *Kobeltostylus*.4Wenz (1939) replaced the name *Salpingophorus* Kobelt & Möllendorff, 1897 by *Annularia* Schumacher, 1817, while Egorov and Greke (2007) regarded both these names as junior synonyms of *Cyclophorus* Montfort, 1810 (see note ^1^).5Cyclophorus (Annularia) aurantiacus is distributed in SE Asia, so that its presence in Nepal is doubtful and requires confirmation.6Nevill (1878) regarded *Scabrina* W. T. Blanford, 1863 as a subgenus of *Cyclophorus* Montfort, 1810. Kobelt and Möllendorff (1897) raised *Scabrina* to genus rank. *Cyclophorus pinnulifer* Benson, 1857 was fixed as the type species of *Scabrina* by Nevill (1878).7Some authors have erroneously attributed the Alycaeinae to Gray, 1850 (Minato 2005, Tarruella and Domènech 2011). However, according to Bouchet and Rocroi (2005) the correct authorship is ‘W. Blanford, 1864’. Alycaeinae comprises four genera (Tarruella and Domènech 2011) namely *Alycaeus* J.E. Gray, 1850, *Chamalycaeus* Kobelt & Möllendorff, 1897, *Cipangocharax* Shintaro, 1934 and *Dioryx* Benson, 1859. The generic names *Alycaeus* and *Chamalycaeus* have been applied by recent authors (Panha and Burch 2005, Maassen 2006, Dumrongrojwattana and Maassen 2008, Lee et al. 2008, Tarruella and Domènech 2011). Conversely, *Cycloryx* Godwin-Austen, 1914, *Dicharax* Kobelt & Möllendorff, 1900 and *Raptomphalus* Godwin-Austen, 1914 are treated as subgenera of *Chamalycaeus* by Gude (1921). We apply Gude’s (1921) generic categories.8The name of the type species of *Alycaeus* J.E. Gray, 1850, by original designation *Cyclostoma gibbum* Férussac, 1838, is a junior homonym of *Cyclostoma gibbum* Draparnaud, 1805 (Hydrobiidae). Therefore, it has been replaced by *Alycaeus eydouxi* Venmans, 1956.9Some authors attribute authorship of Chamalycaeus (Dicharax) strangulatus to Hutton such as Pfeiffer (1846), Nevill (1878), Hanley and Theobald (1878) but without indicating the publication year. Hutton’s name was, however, a manuscript name of no nomenclatural standing. Gude (1921) and Tarruella and Domènech (2011) were correct in assigning authorship to ‘Pfeiffer’.10Seven subgenera have been recognized within *Diplommatina* (Kobelt 1902, Kuroda 1928): *Benigoma* Kuroda, 1928, *Diplommatina* Benson, 1849, *Diploptychia* Möllendorff, 1895, *Metadiancta* Möllendorff, 1898, *Moussonia* Semper, 1865, *Pseudopalaina* Möllendorff, 1898, and *Sinica* Möllendorff, 1885.11Diplommatina (Sinica) canarica is endemic to the Western Ghats (Raheem et al. 2014). Hence, the identification of the Nepalese specimens by Kuznetsov and Schileyko (1997) needs to be verified.12In the original description of *Schistoloma funiculalum* Benson (in Hutton and Benson 1838) distinguished this species from the European fossil species *Cyclostoma mumia* by ‘the rounder and more reflected orange peristome, and by its central position at the base, as well as by the delicate sculpture, and an embossed spiral cord which winds from above the umbilicus to the base, whence the species has received the trivial appellation of ‘Funiculalum’. It is the first known Indian species belonging to pupaeform or subcylindric division of *Cyclostoma*’. Sowerby (1850) changed the spelling ‘*funiculalum*’ to ‘*funiculatum*’ without giving any reason. Gude (1921) considered Benson’s (1838) ‘*funiculalum*’ to be a nomen nudum because of the spelling error (*funiculalum*, laps). Many authors have used ‘*funiculatum*’ (e.g. Gray in Baird 1850, Pfeiffer 1853, Hanley and Theobald 1870, Nevill 1878, Kobelt and Möllendorff 1897, Gude 1921, Ramakrishna et al. 2010). This was, however, an unjustified emendation (ICZN Article 32.2, 32.3) and thus Benson’s name stands. Nepalese specimens differ in possessing a whitish, instead of orange peristome, but the significance of this is unknown.13*Carychium minusculum* Gredler, 1888 was originally described from China, “aus Hupe” (type locality), which is the Chinese Province Hubei (= Hupeh) (See Zilch 1974). The correct publication year is ‘1888’ instead of ‘1887’ as is sometimes mentioned (e.g. Zilch 1974, Nesemann et al. 2007). In Nepal, this species was recorded from two different localities, viz. Kavre District by Nesemann et al. (2007) and Langtang National Park-Syabru by Kuznetsov and Schileyko (1997). PB checked specimens from these two localities (Nesemann’s specimen and an image of Kuznetsov and Schileyko 1997). The shells from these two localities differ by size, shape and sculpture and may be two distinct taxa.14*Carychium* shells collected by PB from Phulchowki, Shivapuri-Nagarjun and Langtang National Parks have very fine and strong radial ribs, as well as slender apical whorls. As such they differ from the shells of Nesemann et al. (2007), which are comparatively smooth and smaller. They are therefore, tentatively regarded as separate taxa. The Phulchowki specimen was also compared with images of Schileyko’s *Carychium minusculum* specimen from Langtang National Park deposited in ZMMU No. Lc-39251 and *Carychium minusculum* in Zilch (1974: Fig. 13). The peristome along the umbilicus region is more or less straight in the Phulchowki taxon, while it is strongly reflected in Schileyko’s *minusculum*.15According to Kennard (1942) the name *Vaginulus alte* was published in 1822, instead of 1821 as is often mentioned in the literature or 1823 as mentioned in Sherborn (1923: 230). The spelling ‘*altae*’ in e.g. Bössneck (2006) and Raheem et al. (2010) is erroneous.16The type locality of *Pupilla annandalei* Pilsbry, 1921 was doubtfully recorded as Ava (Myanmar) in the Indian Museum (Nevill 1878). Pilsbry (1920-21) speculated that the holotype may have been collected in Nepal because he associated it with central Asian species. However, confirmed records are restricted to granite mountains between 2,000 and 2,800 m in northern Pakistan (Pokryszko et al. 2009).17Pilsbry (1920-21: 204) asserted that *Pupa eurina* Benson, 1864 was collected in Nepal. If correct, Benson’s record would be the earliest scientific report of a land snail from Nepal (Budha 2005). Benson (1864: 139) gave the locality in Latin as ‘*ad Tribeni Ghát fluminis Gogra*’, but we have not been able to identify this locality, since ‘Tribeni’ refers to several localities where two rivers meet and ‘ghat’ refers either to a place where cremations take place or to sites where people cross a river along a trail by using locally made wooden boats. Godwin-Austen (1899: 260) expanded on Benson’s locality information ‘the typical specimens were found in the exuviae of the River Gogra at Tribeni Ghat. This river rises in the Tibetan plateau, and these shells may have been brought down thus from far back in the mountain range’. The downstream course of the Karnali river in Nepal is known as Gogra (= Ghaghara) in India immediately after the two branches of Karnali river meet at the Nepal-India border at Katarniya ghat, Uttar Pradesh, India. The confluence of Seti and Karnali river is called Tribeni which is approx. 100 km upstream (north) from the Nepal-India border. There is no clear evidence that William Theobald ever entered Nepal. However, Joseph Hooker, who was among the earliest Europeans to venture into Nepal to investigate its biota, did spend time with Theobald in India (Hooker 1854: 37, 57) and so it is possible that Hooker passed on samples of *Pupa eurina* to Theobald.18Hutton and Benson (1838) attributed the authorship of *Helix humilis* to Hutton, but Gude (1914) and Ramakrishna et al. (2010) incorrectly cite ‘Benson’ while Sherborn (1927: 3062) cited ‘Hutton & Benson’, 1838 as authors.19*Pyramidula kuznetsovi* was misidentified as *Pyramidula humilis* by Schileyko and Kuznetsov (1997). Kuznetsov’s collections were recently reviewed and Schileyko and Balashov (2012) redescribed the samples as a new species.20The correct spelling is ‘*cantorii*’ and not ‘*cantori*’ as some authors mention (e.g. Zilch 1959, Schileyko 1998).21The type locality of *Mirus nilagiricus* (L. Pfeiffer, 1846) is Nilgiris, South India. Although Kuznetsov and Schileyko (1997) reported this species from Nepal, they question whether the Himalayan species belongs to *Mirus*, though without suggesting an alternative generic placement.22Shortly after the description of *Darwininitium shiwalikianum*, Dr. Somsak Panha communicated that he and Dr. Chirasak Sutcharit (both Chulalongkorn University Bangkok, Thailand) noticed the conchological similarity between this species and *Helix capitium* Benson, 1848, type species of the camaenid genus *Ganesella* W.T. Blanford, 1863. Further anatomical and DNA studies are needed to verify whether *Darwininitium shiwalikianum* and *Ganesella capitium* are conspecific. Moreover, the family level affiliations of *Darwininitium* and *Ganesella* remain to be assessed since the Camaenidae may not be monophyletic (e.g. Scott 1996) and the phylogenetic relationships of the Camaenidae are still poorly resolved (Wade et al. 2007). If *Darwininitium shiwalikianum* is related or identical to *Ganesella capitium*, then it does not represent a pseudosigmurethrous orthurethran condition as was originally claimed by Budha et al. (2012). It would also mean that *Darwininitium* Budha & Mordan, 2012 will be a junior synonym of *Ganesella* W.T. Blanford, 1863.23Nordsieck (1973) assigned Nepalese Phaedusinae to the genus *Hemiphaedusa* and this was followed by Raheem et al. (2010). Later, Nordsieck (2002) described the new subgenus *Montiphaedusa* Nordsieck, 2002 of the genus *Cylindrophaedusa* and grouped all Himalayan clausiliids in *Montiphaedusa*.24Although E African *Lissachatina* is distinguished from W African *Achatina* (Bequaert 1950, Mead 1995), both generic names have been applied to this species. This list follows Budha and Naggs (2008) and Raheem et al. (2010, 2014), who used *Lissachatina* at genus level for reasons further explained by Raheem et al. (2014).25*Cecilioides* is the name used in the original description but it has been variously spelled by different authors. Hermannsen (1846) emended it to *Caecilioides*, which was followed by Pilsbry and Tryon (1908–1909) and Gude (1914). *Cecilioides* has been placed on the official list of generic names (ICZN Opinion 335) and all other spellings are invalid.26Only a single *Cecilioides* shell was collected in Nepal (Baitadi District). It measures about 2 mm, has four whorls, and resembles *Cecilioides minuta*.27Many subulinid genera, such as *Opeas*, *Beckianum*, *Leptopeas*, *Lamellaxis* and *Leptinaria* have been confusingly interpreted (Thompson 2011), even if they are conchologically relatively well-differentiated and anatomical data are available for several of them.28Baker (1935) erected *Allopeas* as a subgenus of *Lamellaxis* Strebel & Pfeiffer, 1882.29Schileyko and Kuznetsov (1997) identified a Nepalese specimen as *Allopeas mauritianum prestoni* (Sykes, 1898) from Annapurna Conservation Area. Sykes (1898) original combination was *Opeas prestoni*. Naggs and Raheem (2000) placed ‘*prestoni*’ under *Allopeas*. Pilsbry and Tryon (1906) placed *Opeas prestoni* Sykes, 1898 under Opeas mauritianum (Pfeiffer, 1852) as var. prestoni. Brodie and Barker (2011) and Bouchet and Cosel (1991) also placed ‘*mauritianum*’ under *Opeas*. Some authors assign ‘*prestoni*’ to *Lamellaxis* (e.g. Deisler and Abbott 1984, Nekola 2014). Griffiths and Florens (2006) suggested that *Allopeas mauritianum* is a junior synonym of *Allopeas clavulinum*. The type locality of this species is Mauritius. FN examined the syntype of *mauritianus* and confirmed that it is identical with material identified as *Allopeas clavulinum*. It has been spread by commerce throughout the tropics but its native range is not known (Hanna 1966, Deisler and Abbott 1984).30As for subulinids in general, *Opeas* species have been confusingly interpreted and have been assigned variously to different genera such as *Allopeas*, *Lamellaxis*, *Paropeas* and *Prosopeas* (although this latter may not even be a subulinid) (Naggs 1994).31We follow Bouchet and Rocroi (2005) and regard Glessulinae as a subfamily of the Subulinidae.32The relationships of *Bacillum* are still unclear. Schileyko (1999) placed the genus in the Rishetiinae Schileyko, 1999, together with *Eutomopeas* Pilsbry, 1946, *Tortaxis* Pilsbry, 1906 and *Rishetia* Godwin-Austen, 1920. Based on the half exposed reproductive parts of a specimen labeled as *Bacillum* sp. Godwin-Austen (1920: 7) states ‘The very recent and extended knowledge of the animals of *Bacillum* and *Glessula* shows that the two genera come next to each other....’ (Godwin-Austen (1920: 7). But the same specimen (NHM) from a lot of 3 specimens from Assam, leg. S.L. Hora, Godwin-Austen coll. (Acc. 1830), Reg. 20120113) examined by PB confirmed that it is closer to *Rishetia* than to *Glessula* since it has an elongated flagellum. Because of its truncated columella, and elongately turreted shell, we provisionally retain *Bacillum* in the Glessulinae.33Subba and Ghosh (2008) recorded *Bacillum* sp. from E Nepal without a description or figure.34Although Martens (1860) designated *Cionella gemma* Benson, 1850 as the type species of *Glessula* (e.g. Zilch 1959), the correct type species is *Achatina ceylanica* L. Pfeiffer, 1845 (Gude 1914, Raheem et al. 2014). This is because *Achatina ceylanica* is the type species (by monotypy) of the genus *Electra* Albers, 1850, which is a junior homonym of *Electra* Lamouroux, 1816 (Ectoprocta). Therefore *Electra* Albers, 1850 was replaced by *Glessula* Martens, 1860 and in such cases ICZN Art. 67.8 rules that the type species of the replaced genus name is automatically also the type species of the new genus name.35The type locality of *Glessula subjerdoni* is S India: Jaypore and Golconda Hills (Beddome 1906). Specimens from NE India (Darjeeling) were erroneously identified as *Glessula subjerdoni* by Gude (1914) and were subsequently assigned to *Glessula crassula* (Reeve, 1850) by Godwin-Austen (1920). Nevertheless, later authors (Kuznetsov 1996, Dey and Mitra 2000, Ramakrishna et al. 2010) have followed Gude (1914). Raheem et al. (2014) consider *Glessula subjerdoni* to be a ‘nomen dubium’.36The distribution range of *Rishetia* in this list is based on unpublished anatomical data of specimens from Nepal and Sri Lanka. For example, Dinarzarde Raheem’s unpublished figures of dissected specimen of *Glessula capillacea* (L. Pfeiffer, 1855) from Sri Lanka indicate that it belongs to *Rishetia* because it has an elongated flagellum typical of *Rishetia*.37Specimens of *Rishetia tenuispira* from Nepal were first described under the genus name *Ranibania* Schileyko & Kuznetsov, 1996. *Ranibania* was subsequently synonymized with *Rishetia* (Schileyko, 1999). However, Schileyko’s (1999) *Rishetia tenuispira* (Benson) from Nepal differs from Benson’s *Rishetia tenuispira* from the type locality, Khasi Hills NE India and is similar to Godwin-Austen’s *Rishetia longispira* Godwin-Austen, 1920. Khanal and Budha (2013) identified specimens of the same locality as Schileyko (1999; Balaju, Raniban, Nepal) as Rishetia cf. longispira. Godwin-Austen (1920) gave a very confusing and conflicting account on *longispira* and *tenuispira* (p. 33 same animal characters including reproductive anatomy) but the distribution range of *longispira* was recorded as westward from Bhutan to Sikkim and Darjeeling, whereas *tenuispira* was recorded from the Khasi and Garo Hills (p. 11–12).38*Gulella bicolor* was originally described as *Pupa bicolor* Hutton, 1834 but Blanford and Godwin-Austen (1908) assigned it to *Ennea* H. Adams & A. Adams, 1855. The species has also been included in the Indo-Chinese streptaxid genus *Sinoennea* Kobelt, 1904. DNA sequence data, however, suggest that *Pupa bicolor* comes within *Gulella* (Rowson et al. 2011).39The record of *Endothyrella plectosoma* (Benson, 1836) from Pegu (=Bamo, Myanmar) (Gude 1914: 81) is probably erroneous (Páll-Gergely et al. unpublished manuscript).40Kuznetsov and Schileyko (1997) recorded *Endothyrella affinis* from Swoyambhunath temple forest area, but the material from this area may be a different species (Páll-Gergely et al. unpublished manuscript).41Some authors ‘1852’ as the publication year of *Kaliella barrakporensis* (e.g. Godwin-Austen 1882, Blanford and Godwin-Austen 1908). Raheem et al. (2014) pointed out that part 20 p. 156 of the Proceedings of the Zoological Society of London was published in 1854, 1852 being the date when the proceedings were presented at the Society’s meetings (see Duncan 1937). Therefore this publication was preceded by Pfeiffer’s ‘*Helix barrakporensis* 1853 Monographia Heliceorum Viventium 3: 59’. So the correct publication year is 1853, not 1852.42According to Falkner et al. (2002) *Euconulus fulvus* is a species complex.43The genus *Hawaiia* is assigned to the Vitrinidae by Vaught (1989), to the Zonitidae by Riedel (1980) and to the Pristilomatidae by Anderson (2005). This later placement is followed in this list.44Cockerell (1891, 1893) published the name as ‘*Cryptausteniae*’ (plural), while in 1898 he corrected it to ‘*Cryptaustenia*’ (singular). However, according to Art 11.8 and 33.2.2 of ICZN, the publication date of the corrected name remains ‘1891’.45There is still much nomenclatural and taxonomic confusion with respect to the genus *Macrochlamys* and its type species. This list follows Raheem et al. (2014) in applying the current genus-level interpretation of *Macrochlamys* sensu Godwin-Austen (1883) with *Macrochlamys indica* Benson in Godwin-Austen, 1883 as its type species.46According to Kiauta and Butot (1973) *Macrochlamys tugurium* would be the most common land gastropod of Kathmandu Valley but so far PB has not recorded *Macrochlamys tugurium* in this area. The most common land gastropod in the Kathmandu Valley is *Bensonies nepalensis*, because of its similar shell shape and size, may have been misidentified as *Macrochlamys tugurium*.47Cockerell (1891, 1893) published the name as ‘*Euausteniae*’ (plural), while in 1898 he corrected it to ‘*Euaustenia*’ (singular). According to Art. 11.8 and 33.2.2 of the ICZN, the publication date of the corrected name remains ‘1891’.48The publication date of *Vitrina monticola* L. Pfeiffer is ‘1849’ not ‘1848’ as cited by some authors mention (e.g. Blanford and Godwin-Austen 1908, Schileyko 2003, Mitra et al. 2005, Ramakrishna et al. 2010). See Duncan (1937) for the publication date of the *Proceedings of the Zoological Society of London* part 16:107); see also Sherborn (1928).49*Bensonies nepalensis* shows a remarkable shell colour polymorphism that seems to correlate with altitude: at lower altitudes in C Nepal (Chitwan District) the body whorl of shells shows a dark brown band on a chocolate brown or white background. They co-occur with banded shells which are similar to mid hill specimens (PB, unpublished observations).50*Himalodiscus aculeatus* was originally assigned to the Discidae by Kuznetsov (1996) based on conchological features, but based on anatomical data Schileyko and Kuznetsov (1998b) re-assigned it to Ariophantidae.51The type species ‘*Helix vidua*’ has been confusingly cited. Zilch (1960) mentions ‘*Euplecta vidua* W.T. Blanford’, but Schileyko (2002) mentions ‘*Euplecta vidua* Hanley and Theobald, 1875’. Godwin-Austen (1876) mentions ‘Euplecta (Rotula) vidua Blanford’, whereas Blanford and Godwin-Austen (1908) list ‘*Khasiella vidua* Blanford’ in the same book under its species description as ‘*Khasiella vidua* H. & T. (Blf. MSS) (*Helix*)’. We follow Coan and Kabat (2012) in referring the type species to as *Helix vidua* Hanley & Theobald, 1875.52The identification of Nepalese *Khasiella pansa* needs to be verified.53Schileyko and Frank (1994) and Kuznetsov and Schileyko (1997) reported *Oxytesta orobia* from the neighbourhood of Kathmandu, Nepal. PB checked the syntypes in NHM and specimens available at RBINS and compared these with Nepalese shells and concluded that the Nepalese specimens belong to a different species.54*Sarama* Blanford and Godwin-Austen, 1908 is a junior homonym of *Sarama* Moore, 1887. *Saramina* Wenz, 1947 is a junior synonym of *Rasama* Laidlaw, 1932.55The subgeneric name *Taulimax* Wiktor and Likharev, 1980 is a junior synonym of *Kasperia* Godwin-Austen, 1914 (Wiktor 2001b).56Bössneck (2006) and Raheem et al. (2010) misspelled the genus name as *Turcolimax*.57Although *Anadenus giganteus* Heynemann, 1863 is the type species of *Anadenus* Heynemann, 1863 (Zilch 1959, Wiktor et al. 2000), Wiktor (2001a) proposed to replace it by *Limax altivagus* Theobald, 1862, because he regarded *Anadenus giganteus* Heynemann, 1863 as a ‘nomen dubium’. For the time being, we nevertheless maintain *Anadenus giganteus* as the type species as Simroth (1901) did provide anatomical data, including figures of *Anadenus giganteus*.58Three paratypes of *Anadenus nepalensis* from Langtang National Park in fact belong to *Anadenus altivagus* viz. one specimen from ‘Chandrabar (= Chandanbari), 3,300 m a.s.l., fir forest’ and two specimens from ‘Gosainkund, 4,200 m a.s.l.’ both collected on 27.09.1981 by A. Kuska (see Wiktor 2001a) (A. Wiktor, pers.comm. 13.10.2009). This was confirmed by their reproductive anatomy (with its typical spines inside the penis) examined by PB.59Wiktor et al. (2000) figured the reproductive anatomy of Meghimatium cf. pictum (Stoliczka, 1873) and *Meghimatium bilineatum* (Benson, 1842) based on Chinese specimens but found no clear differences and hence were undecided as to whether or not Meghimatium cf. pictum is a distinct species. The reproductive organs of a specimen from Nepal resemble those of Chinese Meghimatium cf. pictum.60Bradybaena (?) thakkholensis was described on the basis of a few juvenile shells by Schileyko and Kuznetsov (1998a), its anatomy is unknown.61Subba and Ghosh (2008) misspelled this species name as Aegista (Placetotropis) tapeina.62The correct spelling is ‘*huttonii*’ (Raheem et al. 2014; see Pfeiffer 1842: 82), not ‘*huttoni*’ as mentioned by e.g. Zilch (1960) and Schileyko (2004).63*Landouria coeni* was placed in the subgenus *Plectotropis* of the genus *Aegista* by Ramakrishna et al. (2010). However, these two taxa were treated as distinct genera by Preston (1914), Gude (1914), and Schileyko (2004).
